# Cellular apoptosis induced by replication of hepatitis B virus: possible link between viral genotype and clinical outcome

**DOI:** 10.1186/1743-422X-4-117

**Published:** 2007-10-31

**Authors:** Yi Wei Lu, Tuan Lin Tan, Jianhua Zhang, Wei Ning Chen

**Affiliations:** 1 School of Chemical and Biomedical Engineering, Nanyang Technological University, 62 Nanyang Drive, 637459, Singapore

## Abstract

HBV remains one of the major pathogens of liver diseases but the outcomes as inflammation, cirrhosis and cancer of the liver are greatly related to different viral genotypes. The aim of this study was to assess the pro-apoptotic effect of HBSP from three HBV genotypes on liver derived cells. HepG2 cells were applied in our system and transfected by HBV genotype A, B, and C. Cells were observed under phase contrast microscope, stained by apoptosis marker and analyzed by flow cytometre. HBSP expression was detected by western blot assay. BH3 sequences were aligned and analyzed by Vector NTI. HBV genotypes A, B, and C transfected cells displayed evidence of cell death which was further proved as apoptosis. Natural expression of a pro-apoptotic protein HBSP was detected during genomes transfection. The different apoptotic effects were correlated to the HBSP expression from each genome. Alignment and analysis of the BH3 domains from the three genomes revealed slight variance which might also contribute to the result. Our results suggested that variant HBSP expression and BH3 sequence of HBV genotypes may be involved in differential apoptotic effect in transfected cells. Detailed analysis of the role of HBV genotypes in cellular apoptotic process should provide molecular information on the reported clinical outcome of infection by different HBV genotypes.

## Introduction

Hepatitis B virus (HBV), with eight genotypes (A-H) based on sequence divergence, is one of the global health threats with over 400 million people currently infected [1].

Outcome of the infection includes viral hepatitis, liver fibrosis or cirrhosis and ultimate hepatocellular carcinoma (HCC). Genotypes with distinct geographic distribution lead to different clinic manifestations. Genotype B is more inclined to develop HCC, whereas genotype A and C cause hepatitis and cirrhosis more that cancer [2]. Viral hepatitis is characterized by diffused inflammatory reaction and associated with cell damage and death [3]. The mechanisms of cell damage are generally defined as the result of a cytotoxic-T lymphocyte (CTL) mediated immune response against the viral infection [4,5]. Another typical process causing cell death is apoptosis, the programmed cell death [6]. HBV viral proteins, such as HBx and HBSP, have been proved able to induce apoptosis [7,8]. This regulated apoptosis might be the strategies developed by virus in order to maximize the production of virus progeny and promote the spread to neighboring cells. However, HBV was yet confirmed to directly cause hepatocyte death.

It has been reported that the mitochondria-dependent apoptotic pathway which is governed by Bcl-2 family of proteins is involved in the development of liver diseases [9,10]. The Bcl-2 family of proteins is defined as the key regulator of apoptosis in the mitochondria-dependent way. They consist of both suppressors and promoters of apoptosis. Four conserved domains within the Bcl-2 family of proteins have been identified through sequence comparisons and named as Bcl2-homology (BH) domains 1–4, particularly, the BH3 domain promotes cell death in most occasions [11]. Recent reports have identified Bcl2-homology domain 3 (BH3) in HBx and HBSP which cast light on how the HBV viral proteins are involved in apoptosis at molecular level [7,8]. The apoptosis induced by the viral proteins might help the dissemination of viral particles with less host immune neutralization.

In this study we reported evidence of direct cell death caused by HBV genome A, B and C after transfection in HepG2 cells. The transfected cells showed characteristics of cellular apoptosis supported by FACS analysis. Further investigation identified the natural expression of HBSP in HBV genome transfected cells. The observed difference in apoptotic effect caused by the three HBV genotypes revealed different HBSP expression in them. BH3 domain sequence analysis revealed the existence of some variance in the three HBSP proteins which might contribute to the result. The significance of our findings was discussed.

## Materials and methods

### Cell culture and transfection

HepG2 cells (ATCC, USA) were cultured in DMEM (Gibco Dulbecco, Invitrogen Inc., USA) with 5% fetal bovine serum (Invitrogen Inc., USA) and 5% CO_2_. Effectene transfection reagent was applied to transiently expressed proteins in HepG2 cells. The cells were tranfected with plasmids when 50% confluency was reached. Transfected cells were maintained at 37°C and examined according to the experiments.

### FACs assay

Vector pcDNA3.1(+) containing the replicative HBV genome A, B, and C were transiently transfected into 5 × 10^5 ^HepG2 cells, respectively. HepG2 cells transfected with empty vector and cells treated by 50 μM cisplatin for 16 hr were set as (-) and (+) controls. Transfected cells were collected at 24 hr and 48 hr after incubation and analyzed by Apoalert™ annexin-V kit (BD, Biosciences, USA). Cells were rinsed in 100 μl binding buffer and stained with 5 μl annexin-V-FITC and 10 μl propidium iodide (PI). Samples were analyzed on FACS station to determine the apoptotic cell portion after 30 min incubation.

### Western blot analysis

HBSP polyclonal anti-serum was acquired by boosting rabbit using an internal HBSP peptide: CDLNLGQDQQQPVRD (Biogenes, Germany). HBSP protein was detected by primary anti-HBSP antibody in 1:1000 dilution and secondary anti-rabbit antibody conjugated with horseradish peroxidase (Pierce, USA) in 1:5000 dilution. Following ECL detection (Pierce, USA) membrane was developed by Kodak E&D system (Kodak, USA).

### Alignment analysis

HBSP amino acid sequences were analyzed by using Vector NTI9. Result was generated and compared with other BH3 domains.

## Results and discussion

### FACS results

A cell-based system for HBV genome A, B, and C replication was generated by cloning the linearized genome in the vector pcDNA3.1 [12]. It has been shown to produce replicative viral particles into the culture medium [13]. This system was selected to investigate the effect of HBV genomes on the cells.

**Figure 1:**
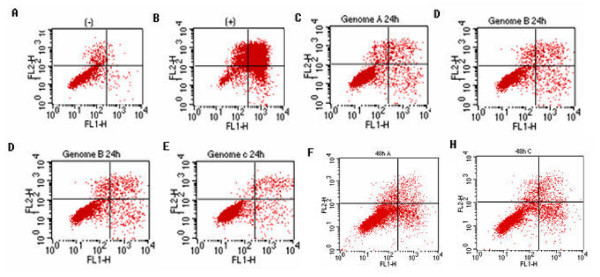
**Flow cytometry analysis of apoptotic effect by HBV genome A, B, and C**. HepG2 cells were transiently transfected with HBV genome A, B, and C and incubated for 24 h (panel C, D, E, respectively) and 48 h (panel F, G, H, respectively) before collected and applied to FACS assay. Cells transfected with empty vector pcDNA3.1 (panel A) and cells treated with cisplatin (panel B) were used as (-) and (+) controls. Cells were labeled by annexin-V-Fitc (FL1-H) and propidium iodide (FL2-H). In each panel, the lower right square (LR) indicates the number of apoptotic cells' portion. Data: LR: A:0.62%, B: 17.32%, C: 2.53%, D: 2.69%, E:1.61%; F: 9.18%, G: 16.13%, H: 6.86%

Recent reports have suggested a new concept that HBV replication is associated with cell death in contrast to the widely accepted non-cytopathic character of HBV [8]. A direct role of viral proteins in apoptosis was also confirmed [7,8,14]. Careful examination of HBV genome A, B and C transfected cells under light microscopy showed rounded up and detached cells which are apoptotic signs. Such morphologies were not observed in normal HepG2 cells and cells transfected with empty plasmid, however, was similar to those cisplatin (a known chemical causes apoptosis) treated cells (data now shown). To identify the observed cell death, FACS was used which is based on the observation that apoptotic cells show externalization of phophatidylserine (PS) on cell membrane [15]. Double staining with FITC (FL1-H) and PI (FL2-H) would indicate apoptotic cells in the bottom-right square of FACS profile as shown in Fig. 1. The results indicated there were more apoptotic cells in the three HBV genomes transfected cells than normal cells at both time point (24 hr and 48 hr). As the incubation prolonged, there was an increase of apoptosis cells in the 48 hr (Fig. F, G, H) samples than the 24 hr samples (Fig 1C,D,E). Specially, genome B displayed a stronger pro-apoptotic effect than genome A and C at both time points (Fig D, G). Our data therefore indicated that HBV genomes were able to induce cell death which is consistant with other viruses capable of inducing cell death. Furthermore, genome B has stronger pro-apoptotic ability than genome A and C.

### HBSP expression

**Figure 2:**
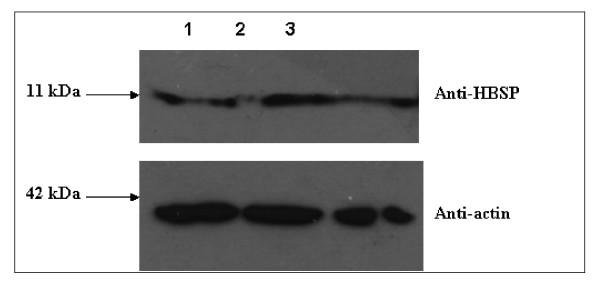
**Natural expression of HBSP in HBV replication**. HBV genome A, B, and C were transfected into HepG2 cells. Cells lysate were detected by anti-HBSP and anti-actin antibodies at 48 hr post-transfection. Lane 1, 2, 3 indicated HBV genome A, B, and C, respectively.

HBSP has been found in HBV infected liver and shown to induce apoptosis through a hitherto unknown mechanism [16,17]. It was also indicated to contain a BH3 domain in the N-terminal and naturally expressed during HBV infection as mRNA of HBSP was detected [8]. However, direct proof of HBSP expression during HBV replication was not characterized. In this study, we used an anti-HBSP antibody (as described in Materials and methods) to detect its expression during HBV genome transfection. To this end, HepG2 cells transfected with the replicative HBV genomes were collected at 48 hr and detected by western blot assay. Results showed detection of HBSP in all the HBV genotypes A, B and C (Fig 2. lane 1, 2, 3). More important, the HBSP expression level in cells showed genome B (Fig 2. Lane 2) had the most abundant HBSP than genome A and C (Fig 2. lane 1, 3). As an internal control, the actin expression in samples was almost equal. This result indicated that the HBSP expression in genotype B was more abundant than those in genotype A and C thus induced higher apoptotic effect as shown in Fig. 1. Our data therefore explained that HBSP was naturally expressed in HBV replication and its expression contributed directly to the observed apoptotic effect caused by HBV genomes.

### Alignment analysis of BH3 domain in HBSPs

**Figure 3:**
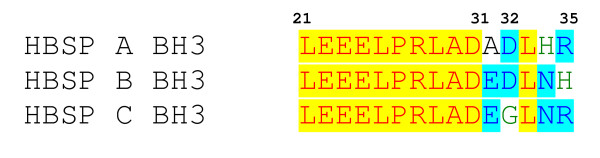
**Alignment of BH3 domains of HBSPs**. Alignment of the BH3 domains revealed the three genotypes share the same conserved amino acid 21–30. BH3 domain is characterized by consensus at position 21, 25, 27, 28, 29, 30, 31 and 32 which are required for interaction. HBSPs differed mainly at position 31 and 32. Genotype B has both of them conserved while A and C has one each (blue color).

As indicated, the HBSP expression might contribute to the viral pro-apoptotic activity. Depending on the slight nuclear acid variance in different genotypes, there raised another possibility that HBSPs might differ in sequence. We aligned the three HBSPs' amino acid sequence and revealed some difference in their BH3 domains (spanning from aa 21–35, Fig. 3). According to the BH3 consensus, the Leu^21^, Leu^25^, Arg^27^, Leu^28^, Ala/Gly^29^, Asp^30^, Glu^31 ^and Asp^32 ^are the most conserved sites [18]. HBSPs shared most of the conserved sites except the acidic aa 31 and 32 which are supposed to be important for electrostatic interaction between Bcl-2 family proteins. Genotype B HBSP possesses both E and D, whereas Genotype A and C have only one each as indicated in blue. This result proposed a possible molecular clue that HBSP genotype B has stronger pro-apoptotic effect than HBSP genotype A and C due to the variance in their BH3 domain. The variance in their HBSP BH3 domain also revealed some clue of different pro-apoptotic property. In BH3 domain, the Leu^21^, Leu^25 ^and Leu^28 ^form the hydrophobic side of the BH3 α-helix to interact with the hydrophobic cleft formed by other anti-apoptotic members of Bcl-2 family. Meanwhile, Arg^27^, Asp^30 ^and Glu^31 ^are important to form electrostatic interaction inside the cleft [18]. Genotype B HBSP is well conserved, including position 31 and 32, while Genotype A has Glu^31 ^and genotype C possesses Asp^32^. This result indicated another possibility how the genotype B caused higher apoptosis.

This study is first to describe HBV genotype A, B, and C lead to apoptosis in HepG2 cell and the slight difference was related to HBSP expression and its property. During the last two decades, it has been widely believed that HBV does not directly cause cell death in host cells [4,5]. Our finding raised the idea that HBV can cause apoptosis with its viral proteins [7,8]. For viruses to avoid the host clearance during the early infection stage they have evolved anti-apoptotic proteins to prevent the host cell from elimination, such as BHRF1 of EBV and E1B19k of Adenovirus [19,20]. On the other hand, viruses also developed pro-apoptotic mechanisms in the late stage of infection to break host cell and promote the spread of viral progeny, like VPR of HIV [21]. It is therefore not surprising that HBV causes apoptosis. HBV chronic infection is considered the main cause of liver cirrhosis and cancer [22]. HBV Genotype B is more related to HCC, whereas genotype A and C are more inclined to cause cirrhosis [2]. This may be related to the severity of persistent HBV infection which determines the infected cell amount. Our finding of the genotype B expresses more HBSP than the other two genotypes and caused higher apoptotic effect supported this hypothesis since it facilitates the spread of viral progeny to infect more healthy cells. Considering the high regeneration capacity of liver cells, it is also possible that an extensive apoptosis would result in a higher level of liver cell proliferation in a regeneration effort. Such an increase in cell division may perturb the normal cell cycle control, resulting in an accumulation of mutations in the genome of progeny cells which ultimately contribute to HCC development. In conclusion, the present study showed the important role of HBSP in HBV induced apoptosis and it determined the variant outcome of different genotypes which might be related to the clinic outcomes.

## Authors' contributions

YWL carried out the experiments on apoptosis and contributed to the first draft of manuscript. TLT contributed in cloning of Bcl-2 family of genes, and helped in Western blot analysis. JZ contributed to cell culture work. WNC initiated the project, interpreted experimental data and finalized the manuscript for submission.
